# Ampullary duodenal diverticulum and cholangitis

**DOI:** 10.1590/S1516-31802003000400007

**Published:** 2003-07-01

**Authors:** Joaquim Mendes Castilho, Manlio Basilio Speranzini

**Keywords:** Duodenal diverticulum, Cholangitis, Choledochojejunostomy, Divertículo duodenal, Colangite, Derivação biliodigestiva

## Abstract

**CONTEXT:**

Ampullary duodenal diverticulum complicated by cholangitis is little known in clinical practice, especially when there are no gallstones in the common bile duct or there is no biliary tree ectasia or hyperamylasemia. A case of this association is presented, in which the surgical treatment was a biliary-enteric bypass.

**CASE REPORT:**

A 74-year-old diabetic white woman was admitted to the Taubaté University Hospital, complaining of pain in the right upper quadrant, jaundice and fever with chills (Charcot's triad). She had had cholecystectomy 30 years earlier. She underwent clinical treatment with parenteral hydration, insulin, antibiotics and symptomatic drugs. Imaging examinations were provided for diagnosis: ultrasound, computed tomography and endoscopic retrograde cholangiopancreatography. The surgical treatment consisted of choledochojejunostomy utilizing a Roux-en-y loop. The postoperative period progressed without incidents, and a DISIDA scan demonstrated the presence of dynamic biliary excretion. The patient remained asymptomatic when seen at outpatient follow-up.

## RESUMO

**CONTEXTO:**

Divertículo duodenal ampolar complicado com colangite é pouco comum na prática clínica, especialmente se há ausência de cálculos no colédoco, ectasia da árvore biliar e hiperamilasemia. Um caso desta associação é apresentado. O tratamento foi cirúrgico por meio de derivação biliodigestiva.

**RELATO DE CASO:**

Mulher branca de 74 anos, diabética, colecistectomizada há 30 anos, foi internada no Hospital Universitário de Taubaté acometida de dor no hipocôndrio direito, icterícia e febre com calafrios (tríade de Charcot). Submeteu-se a tratamento clínico por meio de hidratação parenteral, insulina, antibióticos e medicamentos sintomáticos, enquanto a tomografia computadorizada e a colangiopancreatografia retrógrada endoscópica eram providenciadas para esclarecimento diagnóstico. O tratamento cirúrgico constou de derivação coledocojejunal com alça em Y de Roux. O pós-operatório decorreu sem incidentes; a cintilografia feita com tecnécio-99m (DISIDA) mostrou fácil excreção do contraste para o jejuno e a paciente permanece assintomática em seguimento ambulatorial.

## INTRODUCTION

Most duodenal diverticula are asymptomatic structures. About 75% of duodenal diverticula are located in the second portion of the duodenum. They can be periampullary, when they originate within a range of 2 to 3 cm from Vater's ampulla,^1^ or ampullary when the papilla ends at the bottom of the diverticulum.

The duodenal diverticula rarely produce signs of inflammation, obstruction, hemorrhage or perforation. In some cases secondary biliary-pancreatic complications are found when a diverticulum originates from the region of Vater's papilla. We here describe an ampullary duodenal diverticulum case associated with cholangitis.

## CASE REPORT

The patient, a 74-year-old white woman, was admitted to the Taubaté University Hospital having had pain in the right upper quadrant for 2 days, and jaundice and fever with chills (Charcot's triad). She referred to similar episodes in the past. In giving her history, she revealed that she had been hypertensive for 35 years and was submitted to cholecystectomy 30 years earlier. She had had diabetes over the last 2 years and was taking captopril and insulin daily.

Biochemical evaluations showed: hematocrit 35%, hemoglobin concentration 10.9 g/dl, leukocyte count 30.0 x 10^3^/ml and total bilirubin 3.75 mg/dl, with conjugated bilirubin 3.66 and unconjugated bilirubin 0.9. Serum amylase was 1000 U/l, aspartate transaminase 90 U/l, alkaline phosphatase 818 U/l, gamma-glutamyl transferase 1306 U/l and glycemia 113 mg/dl.

**Figure 1:**
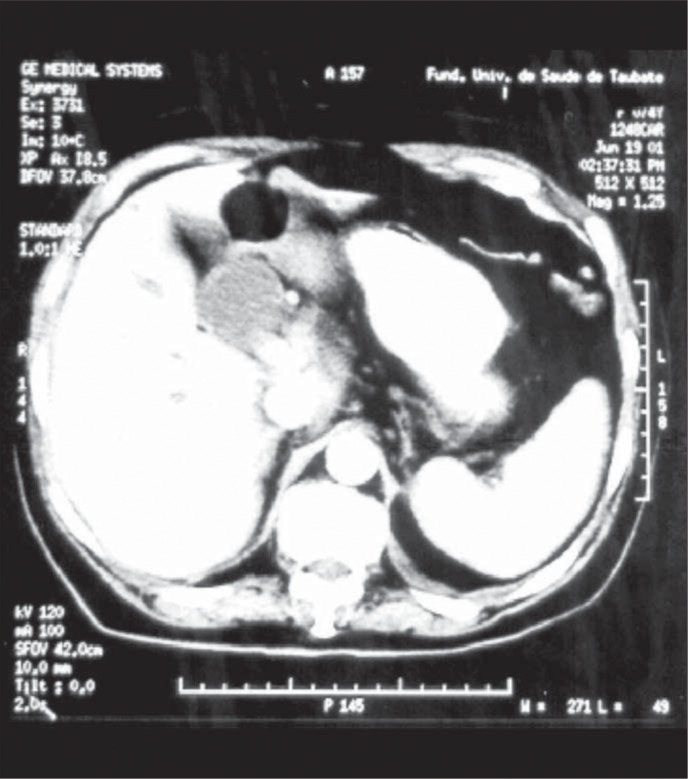
Computed tomography scan of the patient, showing common bile duct that is very much dilated.

**Figure 2:**
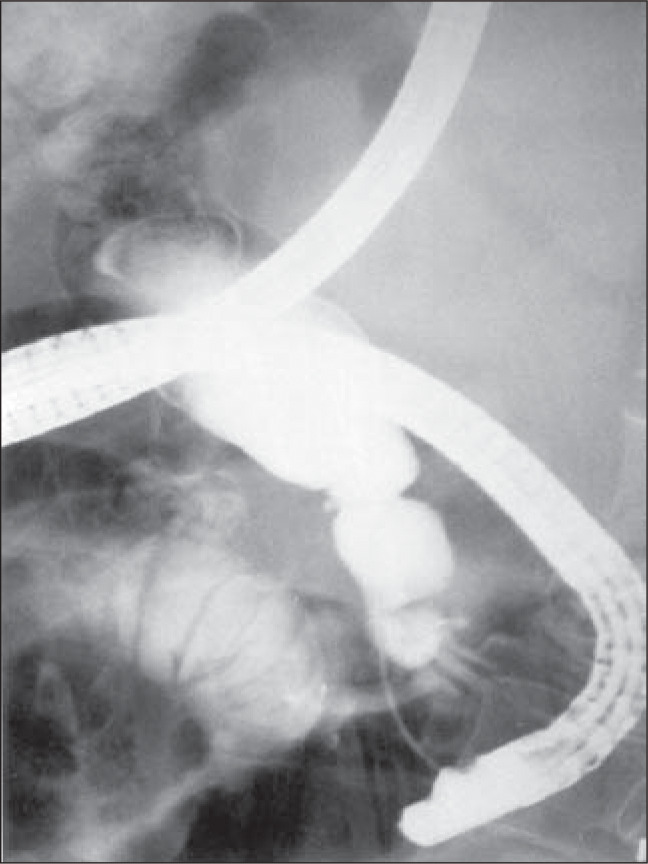
Duodenal diverticulum and absence of gallstones inside the dilated common bile duct, demonstrated by endoscopic retrograde cholangiography.

Abdomen ultrasonography was inconclusive. Computed tomography showed that the intra and extra-hepatic bile ducts were very much dilated and the pancreas head had increased volume and was heterogeneous (Figure 1). Endoscopic retrograde cholangiopancreatography revealed a patent duodenal papilla opening at the bottom of a diverticulum, a common bile duct with an internal diameter of 3.5 cm and the absence of gallstones (Figure 2).

While these examinations were being carried out, the interned patient underwent clinical treatment with hypotensor drugs, insulin, parenteral hydration, third generation cephalosporin (ceftriaxone) and symptomatic medicine. The treatment accomplished provided suggestive regression of the acute-phase symptoms of the disease and consequent decrease in the surgical risk.

The surgery consisted of supra-umbilical median laparotomy to remove the previous scar. The liver was found to have normal size and a smooth surface. Sectioning of the common bile duct was performed at the point of its largest diameter, with distal stump suture and choledochojejunal anastomosis using a Roux-en-Y loop. Choledochostomy was done to evaluate the intraductal pressure, and subsequent cholangiography through a T tube.

**Figure 3:**
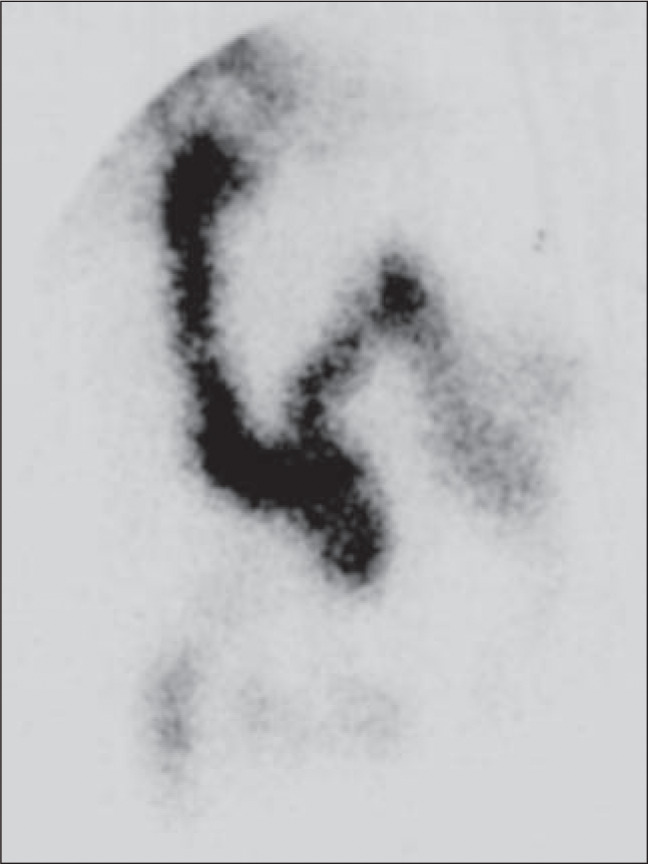
DISIDA scan after biliary-enteric bypass, showing the presence of dynamic biliary excretion.

The postoperative period elapsed without incidents or complications. On the 8^th^ postoperative day, cholangiography through the T tube revealed that the bile ducts were dilated and tortuous, and the choledochojejunal anastomosis was pervious with Roux limb opacification. The patient was discharged one day after this. The drainage tube was removed on the 21^st^ day after the operation. At a follow-up 13 months later, the patient underwent radioisotope scanning using Technetium99m (diisopropylphenyl carboxymethyl iminodiacetic acid, or DISIDA), which showed good excretion into the Roux limb (Figure 3). Postoperative follow-up over approximately two years was uneventful and she continued to present a satisfactory condition.

## DISCUSSION

The ampullary duodenal diverticulum is located at the choledochoduodenal junction and the periampullary diverticulum is at its side. Both types of diverticula are included in the list of possible etiological factors for acute pancreatitis.^2^ According to Naranjo-Chavez et al.,^3^ the ampullary diverticulum is the possible cause of chronic pancreatitis, which would not occur with a periampullary diverticulum. In our report, there was probable pancreatic disease characterized by hyperamylasemia, and the pancreatic head was enlarged with a heterogeneous aspect. The studies of Kim et al.^4^ showed that the association between diverticula and gallstones was significant in patients with primary gallstones but not in those with secondary gallstones. The pathogenesis for the primary stones was associated with the presence of bile stasis and bacterial infection. In our patient, endoscopic retrograde cholangiopancreatography showed ampullary diverticulum, pronounced dilation of the common bile duct, bile stasis and absence of gallstones.

Periampullary diverticula, and particularly the ampullary diverticula, hamper catheterization and bile duct examinations.^5^ Furthermore, giant duodenal diverticula are responsible for the false-positive findings from magnetic resonance imaging, endoscopic retrograde cholangiopancreatography and hepatobiliary scintigraphy.^6^ In the case we report, the papilla was in the diverticulum and catheterization of the common bile duct was performed with difficulty, without direct visualization of the papilla. Oddi's sphincter insufficiency, as verified by endoscopic retrograde cholangiopancreatography, has been reported in the presence of both ampullary and periampullary diverticula.^7^

The cholangitis manifested by our patient can be explained by the increasing bile contamination from bacterial proliferation that takes place in the presence of duodenal diverticula.^8^

Provided that the finding of a diverticulum is only incidental, without signs of inflammation, perforation, hemorrhage or obstruction, no treatment is needed. In cases of biliary-pancreatic disease secondary to a diverticulum, the initial resolution is by means of endoscopic papillotomy, which relieves the jaundice and cholangitis. This is, however, not always definitive due to recurrence over the long term. Surgical removal of the diverticulum, in association with papillosphincteroplasty is the best treatment because it is a simple procedure and avoids recurrence of symptoms in the biliary system.^9^

When diverticulectomy is technically difficult or considered to have major risk in the presence of a dilated bile duct, the best procedure is choledochojejunal anastomosis using a Roux-en-Y loop. For patients with little dilation of the common bile duct or submitted to Billroth II gastrectomy, choledochoduodenal anastomosis may be the procedure of choice.

In the present report, the common bile duct was fully dilated and there were manipulation risks regarding the biliary-pancreatic confluence. Thus, the most appropriate procedure for the patient was to effect total drainage from the common bile duct into the Roux limb of the jejunum, thereby separating the associated link between the diverticulum and cholangitis.

The good postoperative evolution, the DISIDA scan showing good excretion into the Roux limb, and the follow-up with absence of any digestive symptoms, demonstrate the success of the operation.
